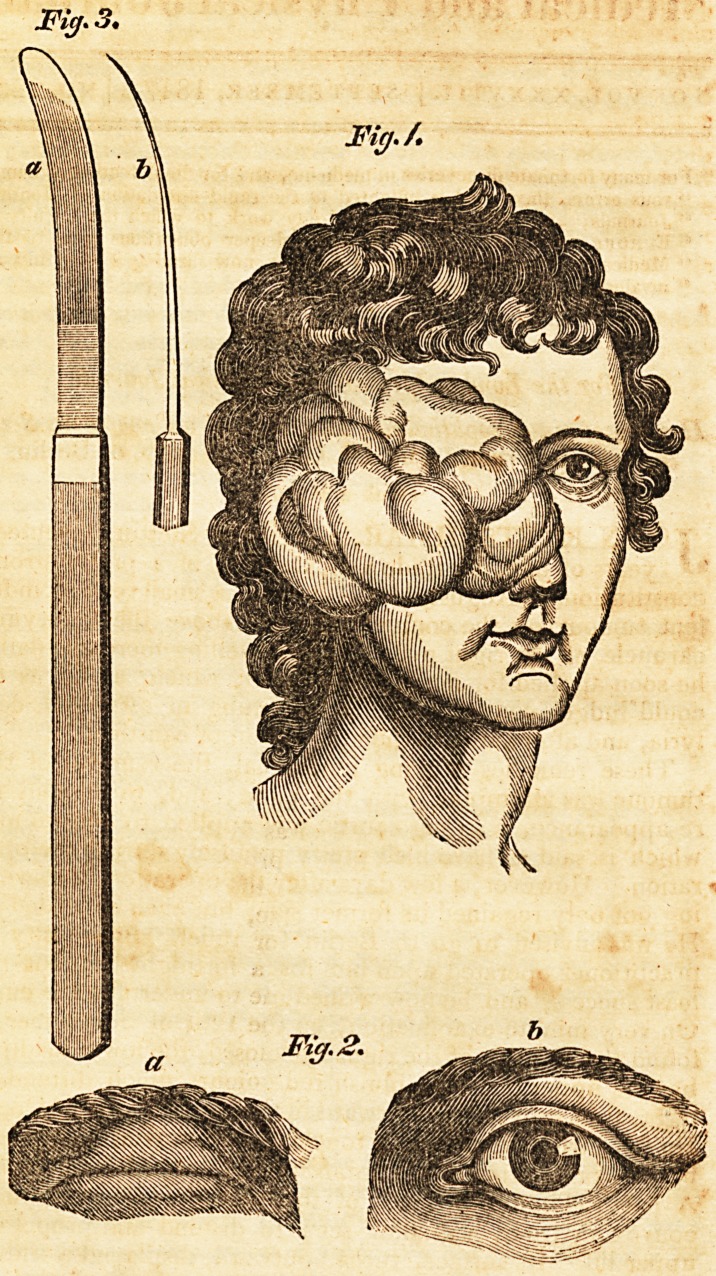# History of an Exophthalmia Fungosa, of an Enormous Size, Successfully Treated

**Published:** 1817-09

**Authors:** G. L. Helling

**Affiliations:** of Berlin.


THE LONDON

### Medical and Physical Journal.

3 OF VOL. XXXVIII.] SEPTEMBER, 1817. [NO. 223.

"For many fortunate discoveries in medicine, and for the detection of numerous errors, the world is indebted to the rapid circulation of Monthly Journals; and there never existed any work to which the Faculty in EUROPE and AMERICA were under deeper obligations than to the Medical and Physical Journal of London, now forming a long, but an invaluable, series."—RUSH.

*For the London Medical and Physical Journal.*

### *History of an Exophthalmia Fungosa, of an enormous Size, successfully Treated;* by Dr. G. L. HELLING, of Berlin.

(With a Plate.)

JOHN KNETSCHMAR, a native of Stettin, seventeen years of age, by trade a lock-smith, of a pretty strong constitution, in August, 1807, observed a small reddish indolent tumour on the conjunctiva, just above the lachrymal caruncle of the right eye. As the swelling increased daily, he soon applied for medical assistance, which, as far as he could judge, consisted in the beginning in astringent collyria, and afterwards in the application of caustics.

These remedies proving ineffectual, the removal of the tumour was attempted with the knife; and, to prevent its re-appearance, a strong caustic was applied to the wound, which is said to have bled pretty profusely during the operation. However, a few days after the operation, the swelling not only regained its former size, but even exceeded it. He was advised to go to Berlin for relief. In this city, a practitioner operated upon him for a fortnight without the least success, and he now wished me to undertake the cure. On very minute examination, on the 12th of September, I found the eye-lids of the right eye closed, the lower healthy, but the upper one of a bluish red colour, much distended, tight, thin, and pressed forward in the form of a pullet's egg. He felt little pain on being touched, but violent if, for the better examination of the organ itself, the eye-lid was raised. A pale red fungous mass, of the size already mentioned, convex in front, was now seen to distend and propel the upper lid. Its surface, turned outward, displayed a trifling elevation and recess, not dissimilar from the convolutions of the brain. The tumour could be raised a little, on doing

 which, which, the cornea was seen underneath in a healthy state, with all the structures behind,—the lachrymal caruncle healthy,—the conjunctiva a little loose, and red toward the bottom, and connected with the tumour upwards, so that it appeared to grow out of the whole conjunctiva along the horizontal diameter of the eye, as far as the ciliar arch, where it had its basis, or rather a stalk, though but a short one, from which it spread to that enormous size. The eye was fixed immoveably downwards and outwards, the sight unimpeded when the tumour was moved sideways, and the rays of light had a free entrance.

The patient recollected no other cause to which to ascribe the disorder but that, about six months ago, in a boxing match, he had received a violent contusion from the finger of his adversary in the interior angle of the eye, which had caused an acute pain in that place. In other respects, he had enjoyed good health from his infancy.

I considered the disorder to be an exophthalmia fungosa, viz. an after-production of the conjunctiva bulbi. On account of its furrowed surface, it appeared to consist, as has already been stated, of several pieces. Of this I convinced myself by separating these various lobes with a blunt probe, which was effected with little hæmorrhage, and without much pain. The conjunctiva had become so loosened as to hang over the cornea in several pieces lying close together, and thus preventing the sight. I had little reason to expect success from any mode of treatment, unless the root of this peduncle was removed. Its base, indeed, was seen in a large circumference, yet, from the direction of the bulb downwards and outwards, and a little forward, it appeared as if the tumour had already extended itself in an inward direction into the orbit, though not deeply backwards, and there drawn other organs into its own diseased state. That the bulb had not been dragged to the place it was in at present by the weight of the swelling, might be concluded, inasmuch as no movement ensued when the conjunctiva was raised, and the bulb thus freed from its burden. The contusion he had suffered six months ago seemed sufficient to produce the disorder in the conjunctiva, which, in that part, is but delicate; nor is it unlikely that the tender os unguis at that time also may have suffered, and that a latent caries in that bone may be considered as the cause which so frequently prevented the removal of the disorder.

Though of opinion that the evil could scarcely be removed, except by removing the eye-ball, yet I resolved to try whether, by removing the front of the tumour, I might gain a better view of the posterior part.

Several

Several physicians with whom I had consulted upon the subject being of the same opinion, I undertook the operation on the 16th of September, in their presence. A strong waxed thread was drawn transversely through the tumour as deep as possible, by means of a needle, from the interior to the exterior angle of the eye, and the ends being tied together, in order to form a hold, by which the tumour was to be drawn from below the upper eye-lid, it was laid hold of by an assistant, another being placed behind the patient, as well for the purpose of holding him as also to raise the upper eye-lid with his fingers, whilst I employed the fingers of my left hand in the same office, in order to acquire a more precise knowledge of those places that were to come under my knife. As the thread seemed likely to break through the tumour, it was found necessary to tie a knot around it, as deep as possible. The tumour being, by this means, pulled more forwards, and the eye-lids more upwards, the former was dissected from the upper margin of the orbit to which it adhered, by means of a convex bistoury; but from the bulb I thought it safer to separate it with the fingers. There was, however, still a place left in the interior angle of the eye, just above the lachrymal caruncle, in which the separation could only be effected by the knife. The tumour was in that place about three lines thicker, and of a more firm, compact, and almost tendinous texture. After the removal of it, the bulb partly resumed its natural situation, its movements being tolerably free, excepting inwards and upwards. All that had the least morbid appearance was carefully removed. This imperfect mobility of the eye made me fearful lest something at the bottom of the tumour might prove a source of re-production. Without, however, entirely extirpating the bulb, it was utterly impossible to go further.

The operation being completed for the present, the patient was put to bed, and, rest being recommended, light compresses, moistened with goulard water and spirit of camphor, were applied to the eye-lids. He suffered so little that he was hardly to be persuaded to keep his bed.

On the 17th of September, the upper eye-lid retracted to its original size.

On the 18th, the patient was serene, and full of expectation of a speedy recovery. I was less sanguine. That part of the conjunctiva from which the tumour had been separated became superficially suppurated.

On the 19th, the tumour began to shoot up near the bulb and the lachrymal caruncle. I now considered caustics the proper application. The kali causticum, with which I in part penetrated pretty deep in the almost insensible tumour, was not only used for several days without advantage, but seemed even to accelerate the growth, so that in twelve days it had already regained its pristine size. The upper eyelid, which before had covered the tumour, and which was very thin, now assumed a different form. It was pushed upwards by the swelling, was œdematous, distended, and adhered to the morbid excrescence in the interior angle of the eye. On the 12th of October, it became inflamed, though it had not been touched with the caustic. The one half, towards the interior angle, containing the lachrymal point, became of a dark red, rather of a bluish cast, and at last black, sphacelous, and dropped off by itself a few days after.

The general health of the patient was little affected. During this time the tumour had likewise increased in size: it was broad in front, but considerably narrower backwards towards the bulb, and moveable, hanging, as it were, on a stalk. The tumour now appeared fit to be tied by a ligature, which I undertook on the 16th of October. The patient complained of little pain from the ligature. In four days, the piece tied came away, but that behind the ligature had gained in size.

I now applied powdered camphor to the morbid growth, strewing it in thick layers, for several days together, but without any benefit, the patient only feeling an increased heat in several places. I then made use of camphor oil, spreading it with a large hair-pencil over the whole swelling, taking care to avoid the neighbouring healthy parts; upon which the surface of the tumour instantly changed its red colour to a white one, forming an eschar, which, to hasten the progress, I removed after a few days with a bistoury, and then spread the oil on again. This time the patient felt likewise no other sensation than an increased heat in the affected part. I repeated this practice several times, but always without effect, for, notwithstanding the repeated removal of the eschar, the fungous tumour soon regained its former size, and even exceeded it.

Nothing seemed now left but the extirpation of the globe: of that operation, however, the patient could not hear, but pressed me to try the ligature again. More with an intention of convincing him of the fruitlessness of a repeated ligature, than in hopes of success, I complied with his request; and, on the 13th of November, applied the second ligature as deep as possible. This time the patient complained of intense pain, which, however, soon ceased. Expecting no other particular symptoms than on the application of the first first ligature, I left him to rest and composure. In a few hours afterwards, however, the pain was insupportable, and extended along the nerves of the upper part of the orbit. The soft frontal parts of the temples and the parotid gland were much swelled. These violent pains were relieved by drawing the ligature tighter. I ordered ten drops of laudanum, and warm fomentations of emollient herbs and of hemlock. The swelling subsided after a day or two. On the sixth day, the 19th of November, I removed the piece before the ligature with the knife, without either pain or hæmorrhage, not waiting for its dropping off, on account of its disagreeable smell.

This ligature proved useless like the first, another fungous mass growing out behind the thread, whilst that in front was coming away. The general health had also suffered; the remainder of the thin upper eye-lid adhered firmly to the tumour, and the whole of the conjunctiva bulbi was connected with it. This morbid growth becoming continually more luxuriant the more it was wounded, I resolved not to meddle with it any farther, representing to the patient the necessity of extirpation if he wished to obtain a radical cure. But neither my reasoning with him nor the proofs I adduced, stating our late disappointment, were sufficient to convince him. Unguentum rosatum, spread on a piece of fine linen, was now the only application. The excrescence increased, however, every day, discharging a fœtid ichor from its dark brown, and in some places white, surface. The pain in the circumference of the orbit grew worse, and, extending to the forehead and temples, disturbed his rest, which reduced the poor sufferer in such a manner, that he became emaciated, lost his appetite, and suffered several fever paroxysms during the nights.

Tired of his sufferings, and excluded from all society on account of the fœtor of the tumour, he returned to me on the 7th of December, requesting some relief. Contrary to my expectations, he had now resolved upon the extirpation, wishing it done as soon as possible. The state of the eye was considerably worse; the cornea, all but a small part opposite the pupil, being covered with ulcers; the whole conjunctiva of the eye under its horizontal diameter had been drawn into the fungous mass, single parts or flaps of it hanging over the lower eye-lid. The lachrymal caruncle adhered so closely to the tumour, now become cancerous, that it could no longer be found. Fig. 1. in the plate is a correct representation of this remarkable tumour, both as to size and shape.

The 9th of December being the day appointed for the operation, operation, I undertook the same in the presence of Drs. Heim, Henry Meyer, Flemming, and Landvoigt, Messrs. Scheel, Meyer, Herrmann, and Helling, surgeons; who all of them agreed with me in my opinion already stated, that the patients' debility could not be better obviated than by a speedy removal of the cause.

I performed the operation in the following manner:—The tumour being pulled upwards by an assistant, I separated the conjunctiva in its connexions from the lower eyelid, with a convex bistoury; then, allowing the swelling to sink downwards, I made a semilunar incision in the upper eyelid close under the ciliar arch, then pulled the tumour with the fingers of my left hand, finally accomplishing the removal of the eye in very little time with my extirpating knife, which for these twelve years I have been in the habit of using on similar occasions.—See the plate, fig. 3, *a* representing the broad surface of the same, and *b* the profile. The lachrymal gland was removed with a simple little hook and the same extirpatory. The pain was only violent for a moment, as the tumour, by its being pulled forth by its weight, already must unavoidably become burthensome to the optic nerve.

The extirpated tumour, together with the bulb, weighed nine ounces; its parenchyma was soft and fungous. The muscles of the bulb and the cellulosa in the anterior angle of the eye, where the excrescence used perpetually to grow forth as often as it was removed, were found affected. The latter formed a hard round stalk, half an inch thick, adhering to the carious part of the os unguis, about three lines in circumference. The hæmorrhage after the operation soon ceased. The orbit was lightly filled up with dry lint, powdered gum arabic being strewed on it, to prevent any subsequent hæmorrhage; over which, a soft compress was laid, and the whole secured by a quadricept head-bandage. The patient was now put to bed, and a little wine, a cordial mixture, with opium, prescribed, on account of his fatigue.

December the 10th.—The patient had had several hours' refreshing sleep, to which he had been for some time a stranger. He took his breakfast with a good appetite.

In this condition he continued, and, on the 18th, the orbit began to fill with healthy granulations.

On the 4th of January, 1808, in the fourth week, the cure was completed, the wound being contracted to a fissure about a quarter of an inch long, (see the plate, fig. 2, *a*,) so as to make the deformity trifling, as may be seen by comparing it with (*b*), representing the healthy eye. For a time the the scar was covered with a soft compress, fastened to the forehead by a silk ribband.

The joy of the late sufferer at being perfectly cured, exceeded all description, as he had given up all hopes of recovery. On the 17th of January he undertook a journey to Stettin, on a visit to his friends, and returned in the latter end of May, five months afterwards, having completed the whole on foot. As I had discharged him on condition of his letting me know should the least unpleasant symptom occur, and have heard nothing of him since, it may be fairly supposed that the cure has proved permanent.

*Berlin; June* 1817.

---

*For the London Medical and Physical Journal.*

## Figures and Tables

**Fig. 3. Fig. 1. Fig. 2. f1:**